# Distinctiveness of genes contributing to growth of *Pseudomonas syringae* in diverse host plant species

**DOI:** 10.1371/journal.pone.0239998

**Published:** 2020-09-28

**Authors:** Tyler C. Helmann, Adam M. Deutschbauer, Steven E. Lindow

**Affiliations:** 1 Department of Plant and Microbial Biology, University of California, Berkeley, California, United States of America; 2 Environmental Genomics and Systems Biology Division, Lawrence Berkeley National Laboratory, Berkeley, California, United States of America; University of Nebraska-Lincoln, UNITED STATES

## Abstract

A variety of traits are necessary for bacterial colonization of the interior of plant hosts, including well-studied virulence effectors as well as other phenotypes contributing to bacterial growth and survival within the apoplast. High-throughput methods such as transposon sequencing (TnSeq) are powerful tools to identify such genes in bacterial pathogens. However, there is little information as to the distinctiveness of traits required for bacterial colonization of different hosts. Here, we utilize randomly barcoded TnSeq (RB-TnSeq) to identify the genes that contribute to the ability of *Pseudomonas syringae* strain B728a to grow within common bean (*Phaseolus vulgaris*), lima bean (*Phaseolus lunatus*), and pepper (*Capsicum annuum*); species representing two different plant families. The magnitude of contribution of most genes to apoplastic fitness in each of the plant hosts was similar. However, 50 genes significantly differed in their fitness contributions to growth within these species. These genes encoded proteins in various functional categories including polysaccharide synthesis and transport, amino acid metabolism and transport, cofactor metabolism, and phytotoxin synthesis and transport. Six genes that encoded unannotated, hypothetical proteins also contributed differentially to growth in these hosts. The genetic repertoire of a relatively promiscuous pathogen such as *P*. *syringae* may thus be shaped, at least in part, by the conditional contribution of some fitness determinants.

## Introduction

*Pseudomonas syringae* is a ubiquitous species complex containing strains commonly found in association with a variety of both healthy and diseased plants. More than 60 pathovars have been identified in this species, where taxonomic placement is primarily associated with their host range on different groups of host plants [1]. The life cycle of *P*. *syringae* is typically considered to consist of two overlapping phases: an initial period of epiphytic growth on the surface of plants followed by subsequent invasion and growth in the intracellular spaces (apoplast) [2]. Under favorable environmental conditions, such as either high humidity or surface moisture and moderate temperatures, large epiphytic bacterial populations can establish on leaves, increasing the likelihood that at least some cells will invade the interior of the leaf [3]. After invasion of the leaf through stomata or openings caused by wounds, rapid bacterial multiplication can occur in plants that are susceptible to infection, resulting in the formation of visible leaf symptoms and eventually localized host cell death [2, 3]. Most attention to traits involved in plant colonization has focused on those contributing to apoplastic growth. Several such traits including the type III secretion system and certain effector proteins, phytotoxins, siderophores, adhesins, and genes contributing to stress tolerance have been identified [4]. While many genes contribute to growth in particular environments, canonical virulence factors such as type III effectors that contribute to the suppression of the host immune system and establishment of an aqueous environment, as well as phytotoxins, are specifically important to growth in the apoplast [5, 6].

Type III effectors in *P*. *syringae* and other bacterial pathogens are important determinants of host specificity since they are often involved in the suppression of plant defenses. Effectors may in turn be recognized by R proteins, resulting in the initiation of defense signaling and host resistance [7]. However, individual type III effector genes can be disrupted with little to no effect on virulence, apparently because most of the many effectors produced by a given bacterium have redundant functions [8]. The linkage between type III effector repertoires and host range however remains unclear [9]. On the other hand, toxins produced by *P*. *syringae* show little host specificity and typically contribute little to its multiplication in plants [10]. Such toxins instead incite characteristic symptoms that follow infection [10]. While the contribution of such traits to the colonization of a given host plant species has been widely documented, most such studies have been limited to a given pathosystem, and we lack an understanding to what extent such traits contribute to bacterial growth in a plant species-dependent manner.

While the identification of fitness factors of plant pathogenic bacteria by classical gene-by-gene disruption studies is a very laborious process, transposon sequencing (TnSeq), whereby a large mixture of insertional mutants is assayed simultaneously, can quantify the contribution of nearly all nonessential genes to growth in a given plant host. We currently lack an understanding of the extent to which genes that contribute to virulence in one such plant species would also contribute to virulence in other potential host plants. Given that many plant pathogenic bacteria such as *P*. *syringae* are relatively promiscuous, growing both on and within a variety of often unrelated plant species [11], they might have evolved and retained virulence genes that are selectively important only in a subset of the plants with which they interact. This important question in the ecology and epidemiology of such species can only be answered by determining the extent to which those traits needed for colonization are distinctive to a given host plant. The global assessment of the contribution of genes of a strain to its success in a myriad of different hosts or habitats was however, until recently, challenging.

Random barcoded TnSeq (RB-TnSeq) is a modification of TnSeq in which 20-nucleotide barcodes are associated with distinct individual transposon insertion sites [12], facilitating the repeated interrogation of the same large collection of random insertional mutants for their contribution to fitness in multiple experiments. Because the location of the transposons in the mutant population only needs to be mapped a single time using TnSeq, the composition of the individual members of the mutagenized community can be easily quantified in each of many subsequent experiments by enumeration of only the amplicon barcode regions (BarSeq) [12]. This technique has been used to identify *P*. *simiae* genes that enable colonization of *Arabidopsis thaliana* roots [13] as well as genes that contributed to competitive growth of *P*. *syringae* B728a on both the leaf surface and in the apoplast of common bean (*Phaseolus vulgaris*) [14]. Combined with *in vitro* profiling to determine the function of these genes, these high-throughput transposon mutant studies provided considerable insight into the various traits required for plant colonization. A variety of non-essential genes were important for growth in the bean apoplast, including those involved in phytotoxin biosynthesis, the type III secretion system, and alginate biosynthesis. In addition to common bean, strain B728a is capable of both multiplication and elicitation of disease symptoms in plant hosts including lima bean (*P*. *lunatus*) and pepper (*Capsicum annuum*) [11]. Since apoplastic colonization requires growth of the bacteria while in intimate contact with host mesophyll cells, we hypothesized that the genes required to colonize these phylogenetically distinct plant species would differ. Because of the distinct metabolites expected within these plants and the likelihood of differential chemical or physical responses to infection, we hypothesized that a non-overlapping set of genes in *P*. *syringae* would contribute to its success in these various hosts. Furthermore, we hypothesized that there would be a greater overlap among those genes that contribute most to growth in common bean and lima bean than to those in pepper due to the close phylogenetic relationship of these two *Phaseolus* species in the Fabaceae family compared to that of *C*. *annuum*, a member of the Solanaceae family, Here, we examine the differential contribution of non-essential genes in *P*. *syringae* strain B728a to its growth in the apoplast of these three plant species.

## Results

### *P*. *syringae* B728a growth is supported in diverse plant hosts

Given that the sensitivity of gene fitness measurements of bacteria interacting with plants using methods such as TnSeq is proportional to the extent of multiplication of the bacteria in the plant [14], we measured the growth of *P*. *syringae* within several possible host plants to determine whether such a strategy could be employed. Population sizes increased approximately 1,000-fold within six days after inoculation in common bean, and over 10,000-fold in both lima bean and pepper (S1 Fig). Such increases indicated that, on average, each inoculated cell had undergone 10 generations of growth in common bean and 13 or more generations of growth in both lima bean and pepper. Lima bean appeared to be relatively more susceptible to infection with *P*. *syringae* than common bean since it enabled more extensive growth of the bacterium within its tissues. In all hosts, infiltration of leaves with bacterial suspensions of 10^5^ cells/ml conferred sufficiently large initial populations of the pathogen (ca. 5 x 10^3^ cells/cm^2^ of leaf) to yield aggregate populations of ca. 10^6^ cells in the collection of several hundred leaves from which cells were subsequently recovered, thus reducing any bottleneck effects. Such initial population sizes were, however, sufficiently low to enable extensive subsequent growth of the inoculated bacterial cells before their recovery from the apoplast.

### Inoculation of a barcoded transposon library in multiple plant hosts

A randomly-barcoded *mariner* transposon library of *P*. *syringae* B728a described previously [14, 15] was inoculated by vacuum infiltration into the leaves of the three plant species to identify genes contributing to host colonization. This mutant library contains 281,417 total strains having insertions mapped in the B728a genome. A subset of 169,826 genic strains in which transposon insertions were in the center 10–90% portion of a given gene, were used for calculating gene fitness values. Fitness contributions could be estimated for 4,296 (84%) of the 5,137 protein-coding genes of this strain. Since the mutant library was cultured in King’s medium B (KB) from a frozen stock immediately prior to plant inoculations, any changes in the proportional representation of transposon mutants in a given gene during overnight growth in this condition were used as the control against which growth of the mutants *in planta* was compared.

**Fig 1:**
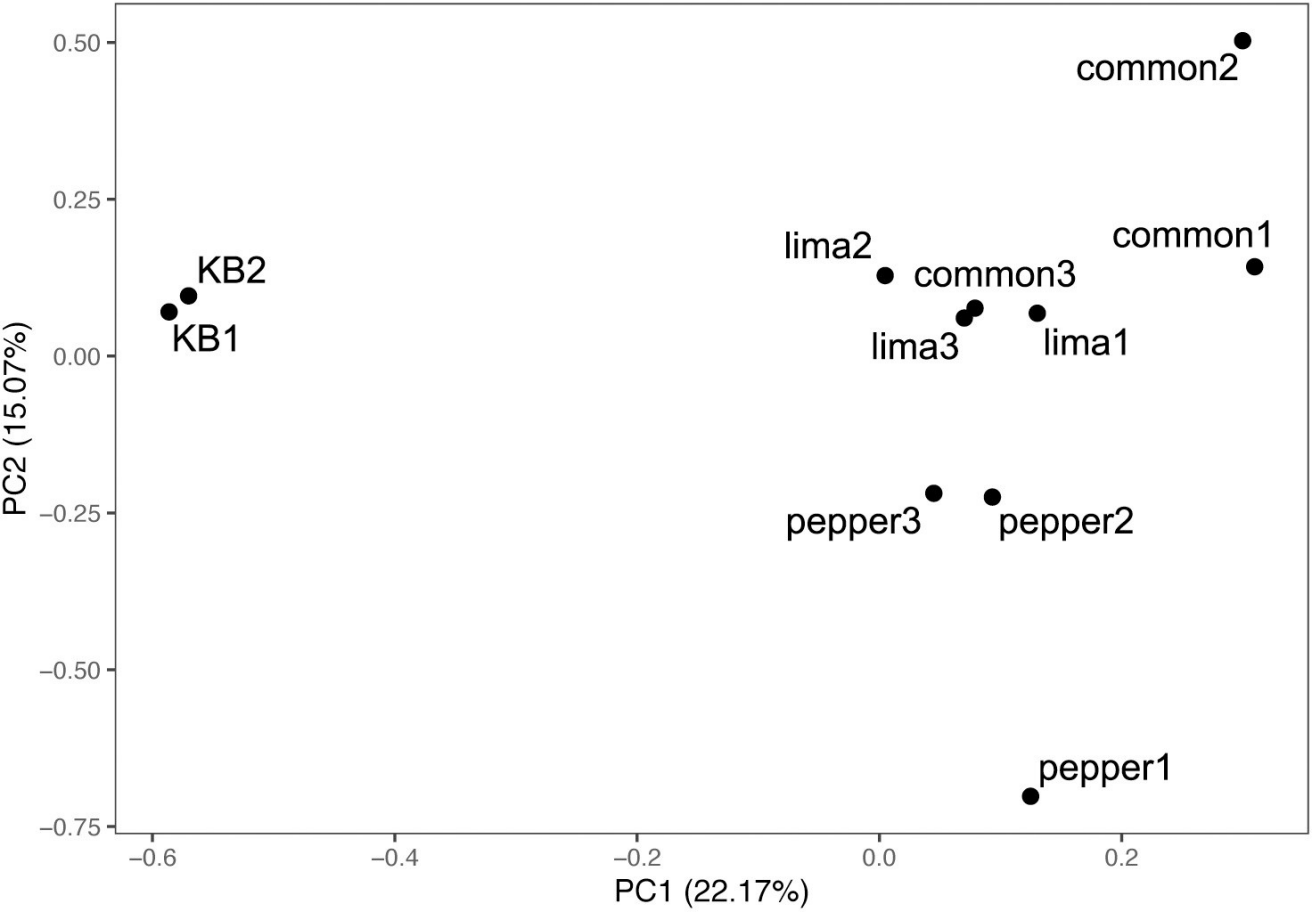
Principle component analysis of *Pseudomonas syringae* B728a grown in King’s B (KB), common bean (“common”), lima bean (“lima”), or pepper. Plot generated using gene fitness values calculated for 4,296 genes. Replicate experiments are noted as the numbers 1 to 3.

Samples of the transposon library recovered from each plant host after growth in the plant for 6 days contained at least 68% the total unique barcodes that were present in the inoculum infiltrated into leaves (S1 Table). This further supports the results of population sizes after inoculation that indicated that a sufficiently large proportion of the transposon mutant population was successfully introduced into plants during inoculation into the apoplast to avoid a large bottleneck. A principle component analysis (PCA) of the fitness data revealed that the contributions of genes to fitness in plants were largely distinct from their contribution to growth in a rich culture medium (Fig 1). The first principle component (PC1) contributed 22.17% of the variance and clearly discriminated between the KB and *in planta* samples. PC2 described 15.07% of the variance and discriminated between pepper and bean. The PCA also indicated minimal differences between the replicates. Despite the differential overall growth in these hosts, fitness values ranged from approximately -5.5 to +1.0 in all replicate studies of these 3 host plants (S2 Fig). For each gene, fitness values for the two replicate growth experiments in KB and the three replicate apoplastic growth experiments performed in each plant host were averaged. While there was variation in the standard deviation of the fitness values estimated for a given gene in a particular host, very few genes exhibited high variation in estimated fitness values and the mean standard deviation did not differ between the three plant species tested here (S3 Fig). It is noteworthy that the standard deviation of estimated fitness values of genes that contributed most strongly to either positively or negatively affecting fitness were generally somewhat higher than those of genes that contributed little to fitness in a given host (S4 Fig), suggesting that the fitness contribution of the traits encoded by these genes was perhaps somewhat context–dependent and thus influenced by idiosyncratic features of particular experiments.

### Common genes for apoplastic fitness in diverse plant species

**Fig 2:**
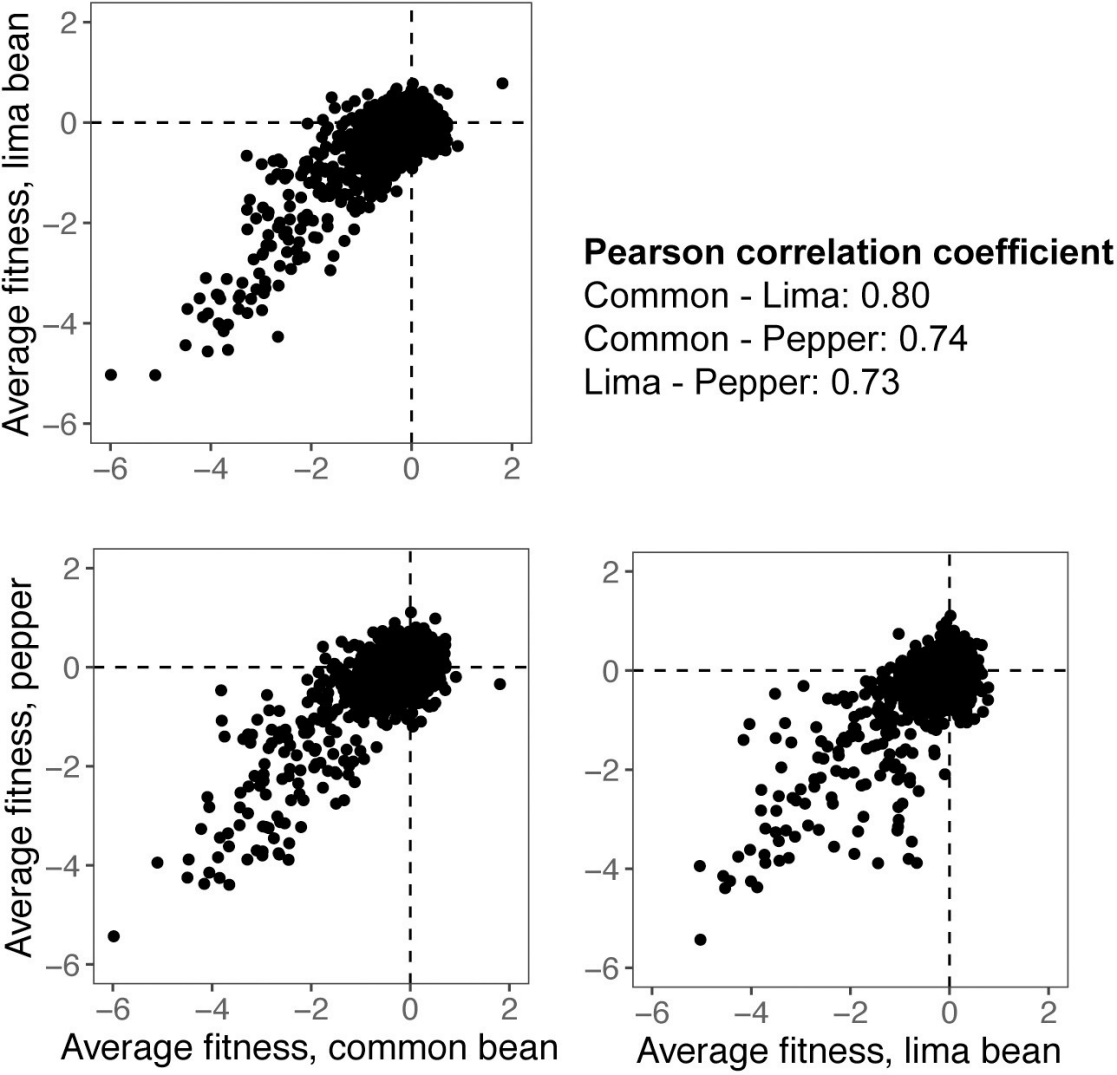
Pairwise comparisons of gene fitness values between plant hosts. There is a high correlation between gene contributions to growth of *Pseudomonas syringae* in the apoplast of common bean, lima bean, and pepper.

**Fig 3:**
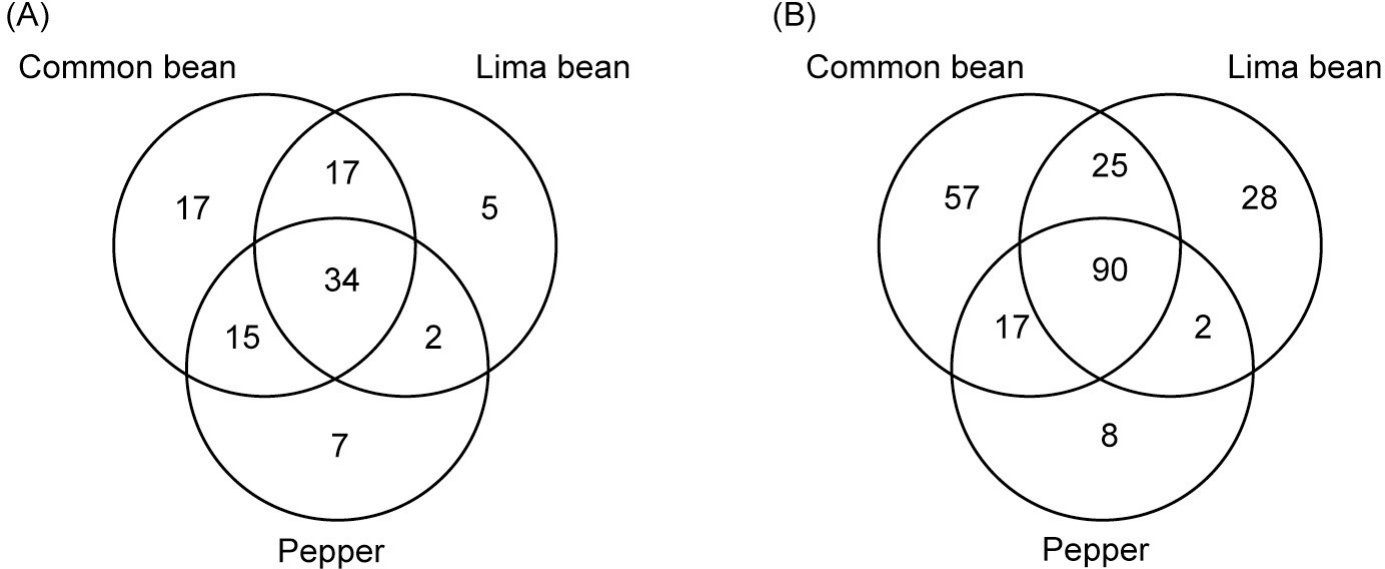
Genes with significant contributions to apoplastic fitness in host plant species. Venn diagrams of the number of genes with average fitness values < -2 (A) or the number of genes with average fitness values < -1 (B).

To determine the extent to which those genes that had been identified as contributing to apoplastic colonization in common bean [14] were also required for maximum fitness in both lima bean and pepper, we compared gene fitness values after inoculating the mutant library into the apoplast of two additional host plant species. Many of the genes of *P*. *syringae* that contributed to its fitness in a given host also contributed similarly to its fitness in the other two plant species tested. Pairwise comparisons of gene fitness values on a given plant species with that in other plant species revealed a high correlation between the fitness contribution of a given gene in the apoplast of common bean, lima bean, and pepper (Fig 2). Pearson correlation coefficients, comparing average fitness values from all genes, ranged from 0.725 (lima bean compared to pepper) to 0.796 (common bean compared to lima bean). While many genes differentially contribute to growth in the apoplast of various plant species, it is clear that nearly as many make similar contributions to fitness on all of the plant species. For both those genes contributing strongly to apoplastic fitness (fitness value < -2) (Fig 3A) or contributing somewhat less to fitness (fitness value < -1) (Fig 3B) approximately 1/3 contributed to competitive fitness in all three host plants tested. Genes with average fitness values below these two fitness thresholds are listed in S2 Table. Those genes with large fitness affects in all hosts are primarily involved in amino acid metabolism, as well as other functional categories including cofactor metabolism, the type III secretion system, and polysaccharide synthesis and regulation (Table 1).

**Table 1:** Number of genes within each functional category with significant contributions to apoplastic fitness in all three host plant species tested.

Functional Category	Fitness < -1	Fitness < -2
Amino acid metabolism and transport	22	15
Unclassified	14	4
Type III secretion system	12	1
Cofactor metabolism	11	0
Polysaccharide synthesis and regulation	6	2
Nucleotide metabolism and transport	4	3
Replication and DNA repair	4	0
Hypothetical	3	2
LPS synthesis and transport	3	2
Carbohydrate metabolism and transport	2	1
Cell division	2	1
Peptidoglycan/cell wall polymers	2	1
Proteases	2	1
Phytotoxin synthesis and transport	1	0
Transcription—Sigma factor	1	1
Transcriptional regulation	1	0
Total	90	34

Fitness cutoffs of < -1 and < -2 are both shown. Functional category annotations are primarily based on COG [16] and KEGG [17] annotations, with manual additions and corrections originally published by Yu et al. [18].

### Differential contributions of genes to apoplastic growth in various plant species

The magnitude of the contribution of 87 genes to apoplastic fitness differed significantly among these 3 plant species (Kruskal-Wallis, p < 0.05). Fifty of those genes had relatively large effects on fitness, having average fitness values of either less than -0.5 or greater than +0.5 in at least one host (S3 Table). Those genes that had a differential impact on fitness in different hosts included eight that were annotated as being involved in polysaccharide synthesis and regulation, six in cofactor metabolism, five in phytotoxin synthesis and transport, and four in amino acid metabolism and transport. We also identified six genes that encoded hypothetical proteins that were differentially important in these hosts. The identity of the other 37 genes that contributed differentially to fitness on these hosts, but whose effect was small, having fitness values greater than -0.5 or less than +0.5 in all host plant species are described in S4 Table.

### Apparent variations in amino acid availability between plant species revealed by fitness of auxotrophic strains

**Fig 4:**
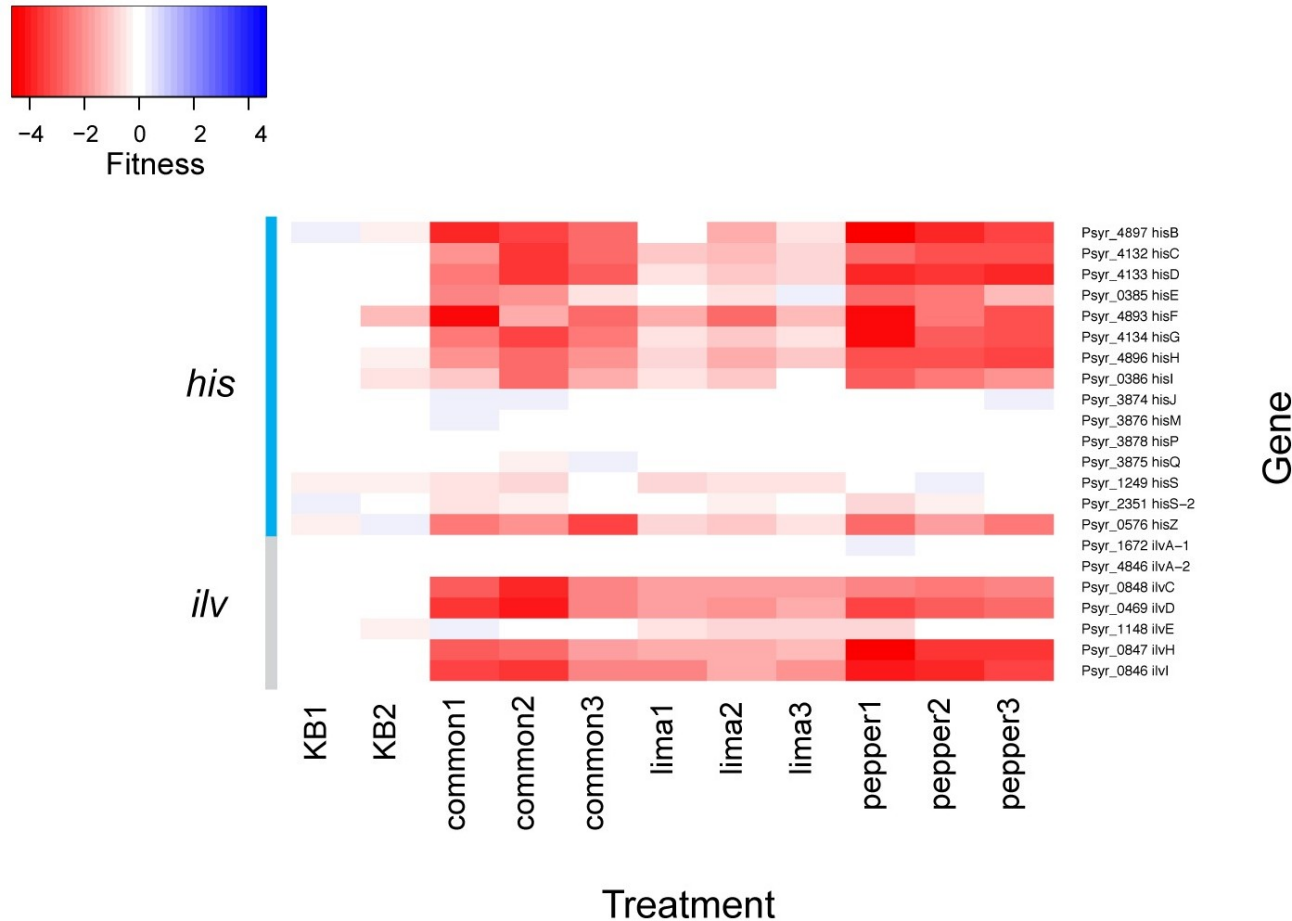
Apoplastic fitness contribution of histidine and isoleucine/valine biosynthetic genes. Genes *hisD*, *hisH*, and *ilvHI* had significantly different fitness values in different plant species by a Kruskal-Wallis test (p < 0.05).

Genes involved in the biosynthesis of several amino acids were generally required for competitive fitness in the apoplast of all plant species tested (Table 1). However, auxotrophic strains in histidine and isoleucine/valine biosynthesis were less fit in common bean and pepper than in lima bean (Fig 4) suggesting that these amino acids were more abundant in lima bean than in the other two species. Within these pathways, average fitness values for *hisD*, *hisH*, and *ilvHI* differed significantly in at least one plant species (Kruskal-Wallis, p < 0.05).

### Phytotoxin production appears to be a host-specific virulence trait

**Fig 5:**
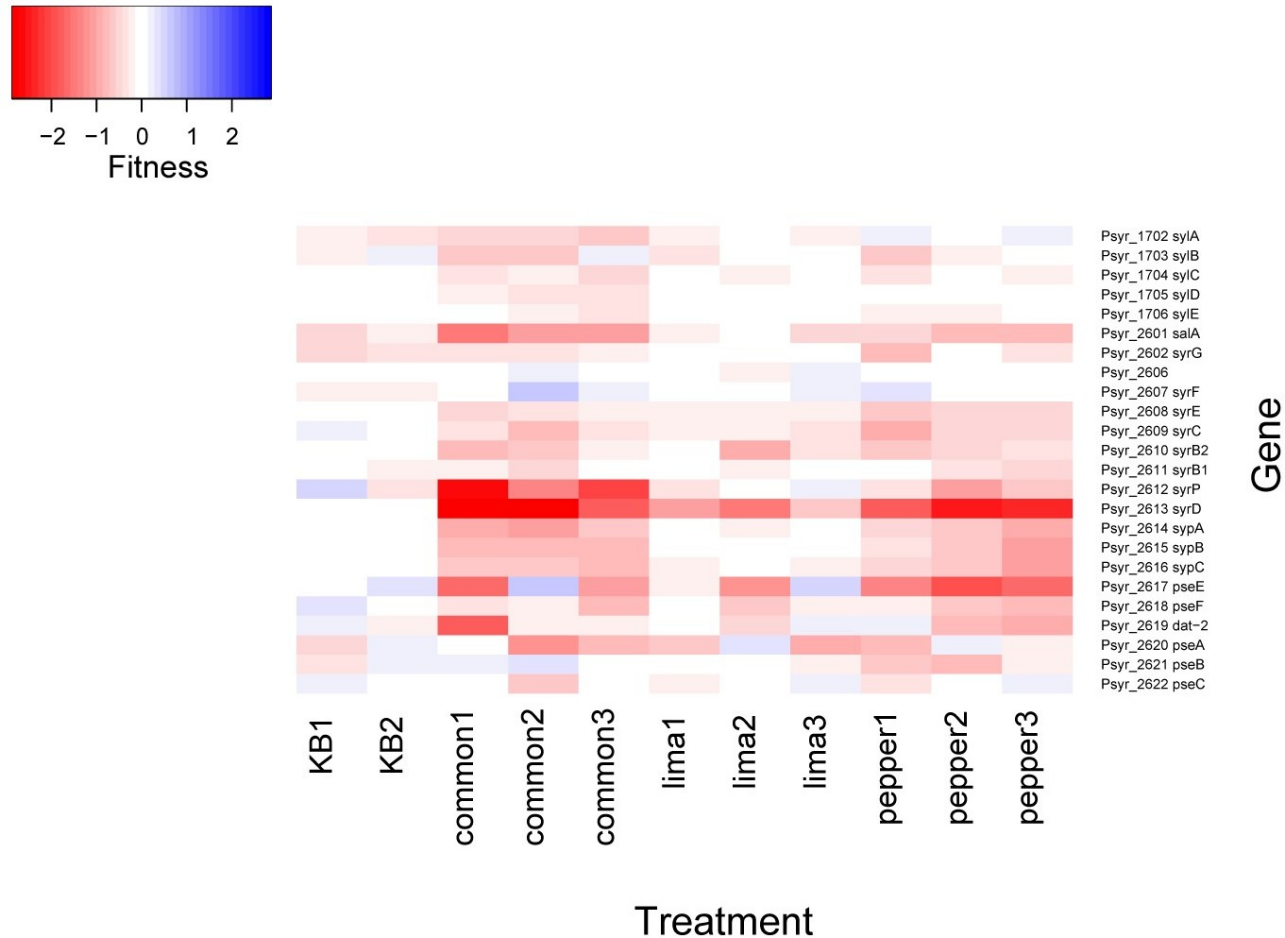
Apoplastic fitness values for genes involved in phytotoxin biosynthesis. Genes *sylA*, *salA*, *syrP*, *sypA*, and *pseB* had significantly different fitness values in these 3 plant species by a Kruskal-Wallis test (p < 0.05).

While the production of phytotoxins such as syringomycin contribute to competitive fitness in the apoplast of common bean [14], it is noteworthy that such genes contributed less to fitness in lima bean than in both common bean and pepper (Fig 5) suggesting that this host was less susceptible to the damage caused by phytotoxins such as syringomycin. The average fitness values of genes encoding the regulatory proteins SalA, SylA, and SyrP, as well as those encoding the secretion protein PseB and the amino acid adenylation protein SypA differed significantly among these three plant species (Kruskal-Wallis p < 0.05).

### Alginate production in *P*. *syringae* varies in importance among host plants

**Fig 6:**
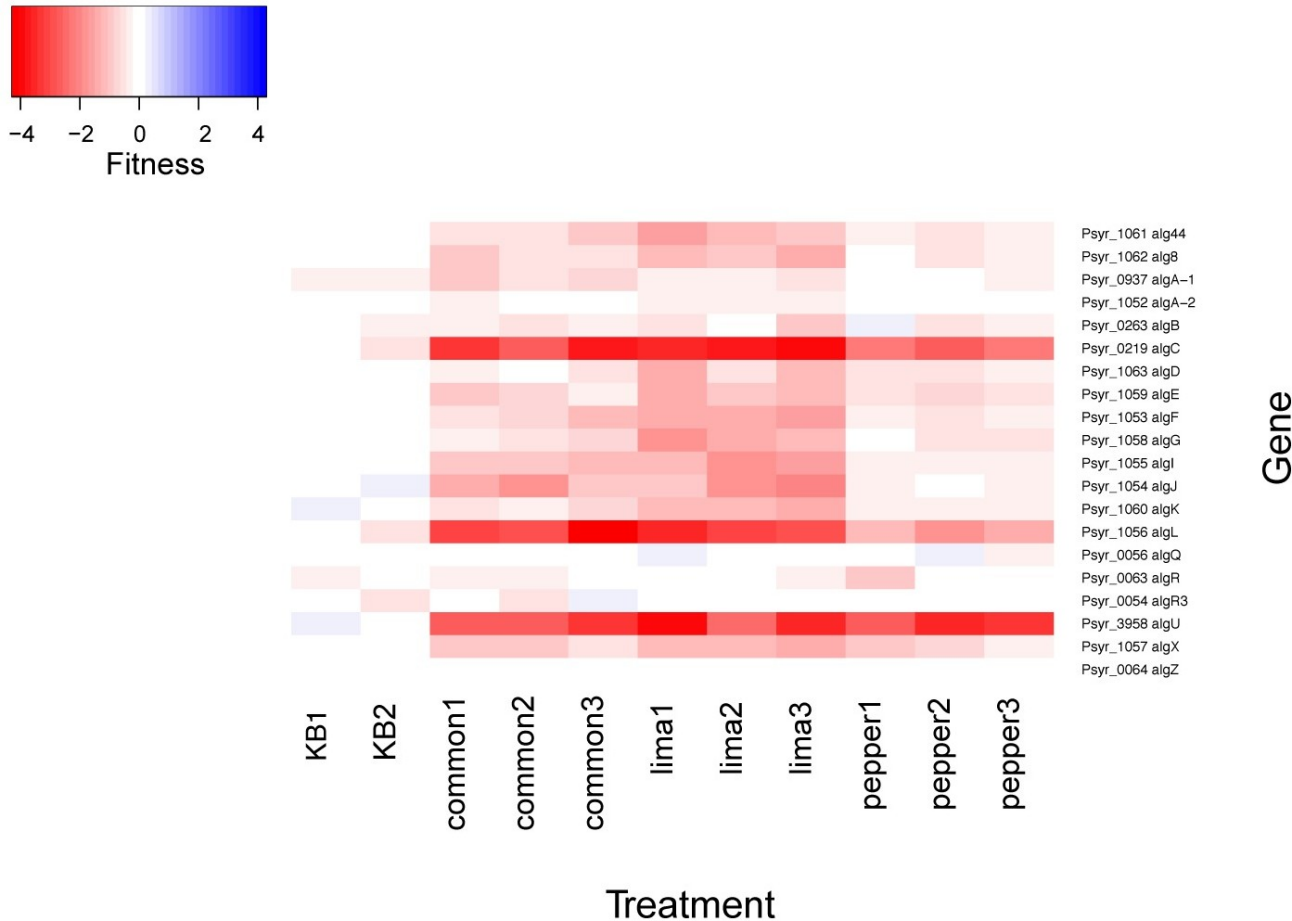
Apoplastic fitness values for alginate biosynthetic genes. Genes *alg44*, *alg8*, *algA-1*, *algF*, *algI*, and *algK* had significantly different fitness values in the 3 plant species by a Kruskal-Wallis test (p < 0.05).

While polysaccharide biosynthesis and regulation is considered a broadly important plant colonization trait, the genes involved in the biosynthesis of alginate contributed differentially to apoplastic growth in the three plant species investigated (Fig 6). Specifically, the alginate biosynthesis genes *alg44*, *alg8*, *algA-1*, *algF*, *algI*, and *algK* were more important in the colonization of lima bean and common bean than in pepper (Kruskal-Wallis, p < 0.05).

## Discussion

RB-TnSeq is a powerful method that has been used in a variety of bacterial species to identify those genes that contribute to fitness in diverse *in vitro* conditions [12, 15, 19]. In one TnSeq study, about 100 genes were found to either increase or decrease the fitness of *Dickeya dadantii* in chicory [20], highlighting the importance of the biosynthetic pathways for leucine, cysteine, and lysine for bacterial growth within the plant. A similar study in *Pantoea stewartii* ssp. *stewartii* identified genes that were important for its growth within corn (*Zea mays*) xylem [21]. Typical of most studies, these reports focused on a single pathosystem. This limited use of this powerful technology is largely due to the cost and effort required to map genomic insertions in the mutagenized population at the start and end of each setting to which the mutagenized population is exposed. In contrast, after initially associating several uniquely tagged transposons with a given gene in RB-TnSeq the relative proportions of the different insertional mutants can be readily assessed by quantifying the amplified barcodes. In this study, interrogation of a mutant mixture after growth in three different host plants provided confident estimates of the differential contribution of each non-essential gene to fitness in these hosts. This proved to be a very powerful tool to generate hypotheses regarding host-specific virulence traits by identifying both common and host-specific genes that enable growth in the intercellular spaces of plants.

We hypothesized that at least a subset of the genes in *P*. *syringae* required for colonization of the apoplast of a given host plant would not be required in other plants, with the assumption that distinctive features of the niche in the interior of these plants would differ in resources and chemical defenses. A larger number of genes are required for colonization of the apoplast of common bean compared to the leaf surface [14], suggesting that the leaf interior provides a more complex chemical or physical habitat than the leaf surface. Through fitness profiling of amino acid auxotrophs, the importance of various amino acid biosynthetic pathways for growth both on the leaf surface and in the apoplast of common bean has been demonstrated. This suggested that some amino acids are less abundant than others in both of these environments, supporting direct measurements made by others [22]. We find here that the relative abundance of available amino acids in the apoplast seems to also vary between plant species. Specifically, the intercellular spaces of lima bean appears to contain more accessible histidine, isoleucine, and valine than in both common bean and pepper since genes for the production of these resources played a lesser role in *P*. *syringae* than in the other plant species (Fig 4). On the other hand, similar relative amounts of other amino acids are apparently present in the three plant species tested, as the genes in other amino acid biosynthetic pathways did not contribute to fitness differentially in plants examined here.

While they are not essential for growth *in planta*, *P*. *syringae* produces various phytotoxins that contribute to symptom development in plants [23, 24]. Strain B728a produces the lipodesinonapeptides syringomycin and syringopeptin, as well as the peptide derivative syringolin A [25, 26]. Syringolin A counteracts stomatal immunity through proteasome inhibition [27], and genes in this biosynthetic pathway did not contribute to apoplastic growth in any of the three plant studied here. Syringomycin and syringopeptin function as pore-forming cytotoxins, contributing to ion leakage [28]. It was interesting to note that the biosynthetic genes for these non-specific toxins contributed more to fitness in common bean and pepper than in lima bean (Fig 5). Curiously, while strain B728a grew to higher population sizes in pepper than in common bean (S1 Fig), symptom development is much less apparent on pepper than in either common or lima bean, without the appearance of any necrosis. While these three plant species were all susceptible to strain B728a, lima bean appeared much more susceptible than common bean because of the substantially higher apoplastic populations that were attained as well as an earlier onset and more extensive necrotic lesion formation that it supported (S1 Fig). Thus, while it appears that syringomycin is a general virulence trait required for maximum growth within plants, and not merely a factor contributing to necrotic lesion development, the mechanism by which it contributes to growth in plants remains unclear. It is possible that differences in the immune responses between these plant species might be modulated by syringomycin, thus contributing to the differential contribution of this trait to apoplastic growth.

Given that many genes contributed similarly to virulence in all three hosts, it remains unclear why *P*. *syringae* strain B728a multiplied to substantially different population sizes in these plants. Low water availability on both the leaf surface and in the apoplast limits bacterial growth [29]. In response, *P*. *syringae* apparently upregulates the production of the exopolysaccharide alginate, to help maintain available moisture for bacterial growth [1]. Alginate production is an important factor for both virulence and epiphytic fitness of *P*. *syringae* [1, 30]. While alginate biosynthetic mutants were less fit than most other mutants in the apoplast of all three hosts tested in this study, alginate appears to be a more important virulence trait in lima bean relative to that in pepper. In *P*. *syringae* pv. *tomato* DC3000, alginate biosynthetic genes are expressed in both host and non-host plants [31]. While alginate production is apparently a generally important virulence trait, differences in its relative contribution to virulence in these plant species may indicate variation in the relative moisture content in the apoplastic space of lima bean and pepper. It is possible that the anatomical differences between these plants contribute to differences in water availability. For example, pepper leaves are thicker than that of either of the two bean species, and its water loss during the infection process may be somewhat less than in lima bean, thus enabling its leaves to better retain water that may be released by the plant to the apoplast during the infection process, diminishing a requirement for alginate production by *P*. *syringae* to maintain hydration [6].

Given that *P*. *syringae* strain B728a is capable of symptom formation in several additional plant species beyond those tested here [11], it should prove informative to interrogate the contribution of both the common and unique virulence genes identified here in a more disparate collection of plant species that presumably would provide yet more distinctive plant habitats that would require additional host-specific fitness traits than those revealed here. Further studies of interactions with the plant species examined here should also enable the identification of additional genes that play a quantitatively smaller, but ecologically important role in their colonization. The fitness values observed for many genes reflected 50% or less growth of the disruption mutants compared to the wild-type strain. Such a large effect of these genes on growth over the relatively few (10 to 13 generations) in these studies suggests that there is strong selection for these genes in plants, especially when considering the many generations that the species would undergo in a given year. Because of the limited number replications used in the study we did not use a multiple testing correction for the Kruskal-Wallis tests because the resulting p-value (10^−5^) was deemed to be too conservative. This risks the identification of some genes as “host-specific” when they were not. However, it is important to note that all of the genes that contributed differentially to colonization of these three plant species (S3 Table) were found to be required for fitness in at least one of these plant species, having an average fitness value less than -0.5 in at least one host. There is less support for a few other genes for which differential contribution fitness was indicated (S4 Table). The contribution of such genes with lesser effects on virulence might be better quantified in further studies in which the mutant mixtures are recovered after infection events and used to re-inoculate additional plants, thereby increasing the number of generations of multiplication over which fitness values could be estimated.

*P*. *syringae* is a commonly observed phyllosphere resident that is also found in other location in the global water cycle [32]. As such, it presumably interacts with a large number of different plants, both as a surface colonist and after entering into plants [33]. These various habitats presumably require very different genes to enable it to exploit these diverse settings. There is therefore much to learn about habitat-specific contributions of its many genes. The high-throughput analysis of insertional mutant mixtures used here not only reveals those traits important in a given setting, but also elucidates the limiting factors in those habitats. As such, further exploitation of RB-TnSeq enables functional genomics to be applied to *P*. *syringae* in its diverse natural habitats. While we considered here the contribution of genes to fitness in several of the many plants in which *P*. *syringae* might find itself, there are many other settings in which a subset of its genes may become particularly important to fitness. Clearly, *P*. *syringae* has maintained genes preferentially important in some hosts, and many other genes may play similar habitat-specific roles.

## Materials and methods

### Bacterial strains and growth media

*P*. *syringae* pv. *syringae* B728a was originally isolated from a bean leaf (*Phaseolus vulgaris*) in Wisconsin [34]. The complete genome for B728a is available on NCBI GenBank as accession CP000075.1 [25]. B728a was grown on King’s B (KB) agar or in KB broth [35], at 28˚C. When appropriate, the following antibiotics were used at the indicated concentrations: 100 μg/ml rifampicin, 100 μg/ml kanamycin, and 21.6 μg/ml natamycin (an anti-fungal). Strains used in this study are listed in S5 Table.

### Plant growth conditions

Common bean (*P*. *vulgaris* var. Blue Lake Bush 274) and lima bean (*P*. *lunatus* var. Haskell) were grown in Super Soil (Scotts Miracle-Gro), at a density of five to seven plants per 10 cm diameter pot, in a greenhouse for two weeks before inoculation. Pepper (*C*. *annuum* var. Cal Wonder) were grown in 10 cm diameter pots (three to five plants per pot) containing Super Soil and grown in a greenhouse for ca. 6 weeks to a height of ca. 30 cm. Leaves were kept dry to minimize epiphytic bacterial populations. Metal halide lights (1000 W) were used to provide supplemental lighting for a 16-hour day length.

### Bacterial apoplastic growth measurements

Wild type strain B728a was grown overnight on KB with rifampicin, washed in 10 mM KPO_4_ (pH 7.0), and adjusted to a cell concentration of 2x10^5^ CFU/ml in 1 mM KPO_4_. Cells were inoculated into leaves using a blunt syringe. Leaf samples (3 discs per leaf) were excised using a 5 mm-diameter cork borer and placed into microfuge tubes containing 200 μl 10 mM KPO_4_ and two 3 mm glass beads, and macerated by shaking for 30 seconds at 2400 rpm in a Mini-Beadbeater-96 (Biospec Products) before dilution plating of appropriate serial dilutions on KB containing rifampicin and natamycin. Colonies were enumerated following two days growth at 28˚C.

### Library recovery and growth in KB

For each inoculation, a 1.25 ml aliquot of a glycerol stock containing the transposon library that had been stored at -80˚C was placed in 25 ml fresh KB with kanamycin and grown for approximately 7 hours at 28˚C with shaking until the culture reached mid-log phase (OD_600_ 0.5–0.7). Time0 samples enumerating the relative abundance of each mutant were collected from the inoculum; 1 ml aliquots were pelleted by centrifugation and the pellets frozen until DNA purification. The remaining cells were then washed twice in 10 mM KPO_4_ prior to plant inoculation.

To determine the growth of the mutant library in KB, a 50 μl log phase cell culture (OD_600_ 0.5) was inoculated into 950 μl KB with kanamycin in a 24-well plate. The plate was incubated at 28˚C with shaking for 15 hours. Cells were collected by centrifugation, and frozen prior to DNA purification. Calculation of the fitness values from the KB control treatment is discussed in [14].

### Inoculations of the transposon library into plants

Washed cells were re-suspended to a concentration of 2x10^5^ CFU/ml in 1 mM KPO_4_. The soil of potted plants was covered with cotton to hold the soil in place, and the foliage was inverted into open containers containing ca. 1.5 L of bacterial cell suspension in an open glass bell jar. A vacuum was then applied to the plants sealed within the bell jar for 1.25 minutes and then removed rapidly to force the inoculum into the evacuated apoplast. Ca. 100 pots of plants of a given species were inoculated for a given replicate experiment. Plants were allowed to dry overnight and then moved to the greenhouse for six days.

### Bacterial isolation from the apoplast

Leaves of each plant species were excised from the plants and then chopped in a blender to yield fragments with an average diameter of about 1 to 4 mm. The slurry of leaf fragments was then placed in a water-filled glass dish and placed in a sonication water bath (Branson 5510, output frequency 40 kHz) for 15 min to remove cells. The resulting slurry-containing cells that were dislodged from the leaf interior was filtered through a coffee filter to remove most plant debris. 10% of the ca. 5–10 L of buffer containing the largely plant-free cell suspension was then subjected to sequential additional filtration steps of Whatman filters (20 μm, 10 μm, and 6 μm) to remove much of the remaining plant debris. Bacterial cells were then removed from suspension by centrifugation at 4696 x g for 10 minutes. The pellet was re-suspended in water, and aliquots of cell pellets were frozen prior to DNA purification.

### DNA isolation and library preparation

DNA from frozen pellets was isolated using the Qiagen DNeasy Blood & Tissue Kit according to manufacturer’s instructions. Cell lysis was done at 50˚C for 10 minutes as per optional instructions. For those samples having excess residual plant material, lysed cells were centrifuged at 1,500 x g for 5 minutes before loading the supernatant onto purification columns. Purified genomic DNA was measured on a nanodrop device and 200 ng of total DNA was used as a template for DNA barcode amplification and adapter ligation as established previously [12]. For each time0 and plant experimental sample, two separately purified DNA samples were sequenced as technical replicates.

### Sequencing and fitness value estimation

Barcode sequencing, mapping, and analysis to calculate the relative abundance of barcodes was done using the RB-TnSeq methodology and computation pipeline developed by Wetmore *et al*. [12]; code available at bitbucket.org/berkeleylab/feba/. Primers used for TnSeq and BarSeq are described in [12]. TnSeq was used to map the insertion sites and associate the DNA barcodes to these insertions, as described in [14]. Unique barcoded mutants were identified as those that both mapped to the genome and for which 3 or more reads were obtained during sequencing of the amplified barcodes in a given experiment. For each experiment, fitness values for each gene were calculated as the log_2_ of the ratio of relative barcode abundance following library growth in a given condition divided by relative abundance in the time0 sample. Fitness values were normalized across the genome so the typical gene had a fitness value of 0. Fitness values from sequencing replicates were averaged for each experiment. All experiments passed previously described quality control metrics [12], with the exception of one technical replicate (*P*.*vulgaris*_2), which was removed from analysis. The requirements for a successful experiment included the detection of more than 50 median reads per gene and a consistency in the calculated fitness value estimated from mutants with insertions in the 5’ and 3’ distal halves of a given gene [12]. Experimental fitness values are publically available at fit.genomics.lbl.gov. Fitness values calculated for growth in common bean were previously generated [14].

### Genomic fitness data analysis

All analysis of gene fitness values and gene metadata was done in R [36]. A PCA plot of experiments was generated from the matrix of gene fitness values using the function prcomp. To better classify genes based on their genomic annotation, we assigned gene names, gene product descriptions, and broad functional categories based on the previously annotated genomic metadata [18]. For each gene, fitness values for experimental replicates were averaged to calculate an average gene fitness value for each plant species. A Kruskal-Wallis test was used to identify those genes in which the median fitness in at least one plant host was significantly different than that in other plant species (p < 0.05). Fitness values in KB were not included in this analysis. From these results, genes were removed to a separate list if the absolute average fitness was less than 0.5 in all conditions. Graphs were plotted in R using the ggplot2 package, version 3.1.1 [37]. Heatmaps were plotted in R using the gplots package, version 3.0.1.1 [38].

## Supporting information

S1 Fig Growth of *P*. *syringae* B728a in the apoplast of the susceptible hosts common bean (*Phaseolus vulgaris*), lima bean (*P*. *lunatus*), and pepper (*Capsicum annuum*). The vertical bars represent the standard deviation of the mean.
(PDF)

S2 Fig Rank ordered mean gene fitness values for each plant species and the KB control medium in which *P*. *syringae* was grown. Fitness values for independent replicate experiments are shown in grey, while mean fitness values are shown in black. Gene fitness value is calculated as the log_2_ of the ratio of the barcode counts following growth in a given setting compared to the barcode counts before inoculation. Black lines indicated at fitness values of -2 and +2 are used to reveal strong phenotypes; for example a value of -2 indicates that mutants are 25% as fit as the typical strain in the mutant library. In each dataset, fitness values < -2 or > +2 are more than three standard deviations from the mean (approximately 0).
(PDF)

S3 Fig Distribution of standard deviation of gene fitness values for each gene calculated from three replicate *in planta* experiments. These distributions are similar for the three plant species.
(PDF)

S4 Fig The standard deviation for estimates of gene fitness values is highest at both high and low measures of gene fitness contribution. For each host plant, the standard deviations of gene fitness values are ranked by average gene fitness, and are highest on average for genes with very low or high fitness value. A generalized additive model (GAM) was used to fit the regression lines.
(PDF)

S1 Table Numbers of unique barcodes per sample and median reads per gene. Values obtained from mid-log phase cultures following library outgrowth and before inoculation (time0) and from leaves pooled after growth in 100 pots of plants of a particular host (sample). Unique barcodes were calculated as the total that mapped to the genome and for which 3 or more reads were obtained in a given experiment. The total number of genome-mapped barcodes in the library was 281,417. Technical (sequencing) replicates are listed separately (“a” and “b”), and share the same time0 reference sample. For an experiment to pass quality control, the median reads per gene in the sample must be ≥ 50 [12].
(DOCX)

S2 Table Unique and shared gene loci among the three hosts tested having average fitness values less than -2 or -1. The fitness value cutoff of < -1 is inclusive of the cutoff < -2, although individual genes may appear significant in different categories. These totals are shown as Venn diagrams in Fig 3.
(DOCX)

S3 Table Genes having a differential fitness contribution to growth in three plant species by a Kruskal-Wallis rank sum test (p < 0.05). Fitness values in KB are included for comparison. This table does not include 37 genes whose average fitness values differed between plant species but whose effect was small, having fitness values greater than -0.5 and less than +0.5 in all host plant species.
(DOCX)

S4 Table Genes having a differential fitness contribution to growth in three plant species by a Kruskal-Wallis rank sum test (p < 0.05), but whose effect was small, having fitness values greater than -0.5 and less than +0.5 in all host plant species. Fitness values in KB are included for comparison.
(DOCX)

S5 Table Strains used in this study. (DOCX)
