# Supplementary figures and images for: Distinctiveness of genes contributing to growth of Pseudomonas syringae in diverse host plant species
Source: PLoS One. 2020 Sep 28;15(9):e0239998. doi: 10.1371/journal.pone.0239998 (PMC7521676; doi:10.1371/journal.pone.0239998)

**S1 Fig**

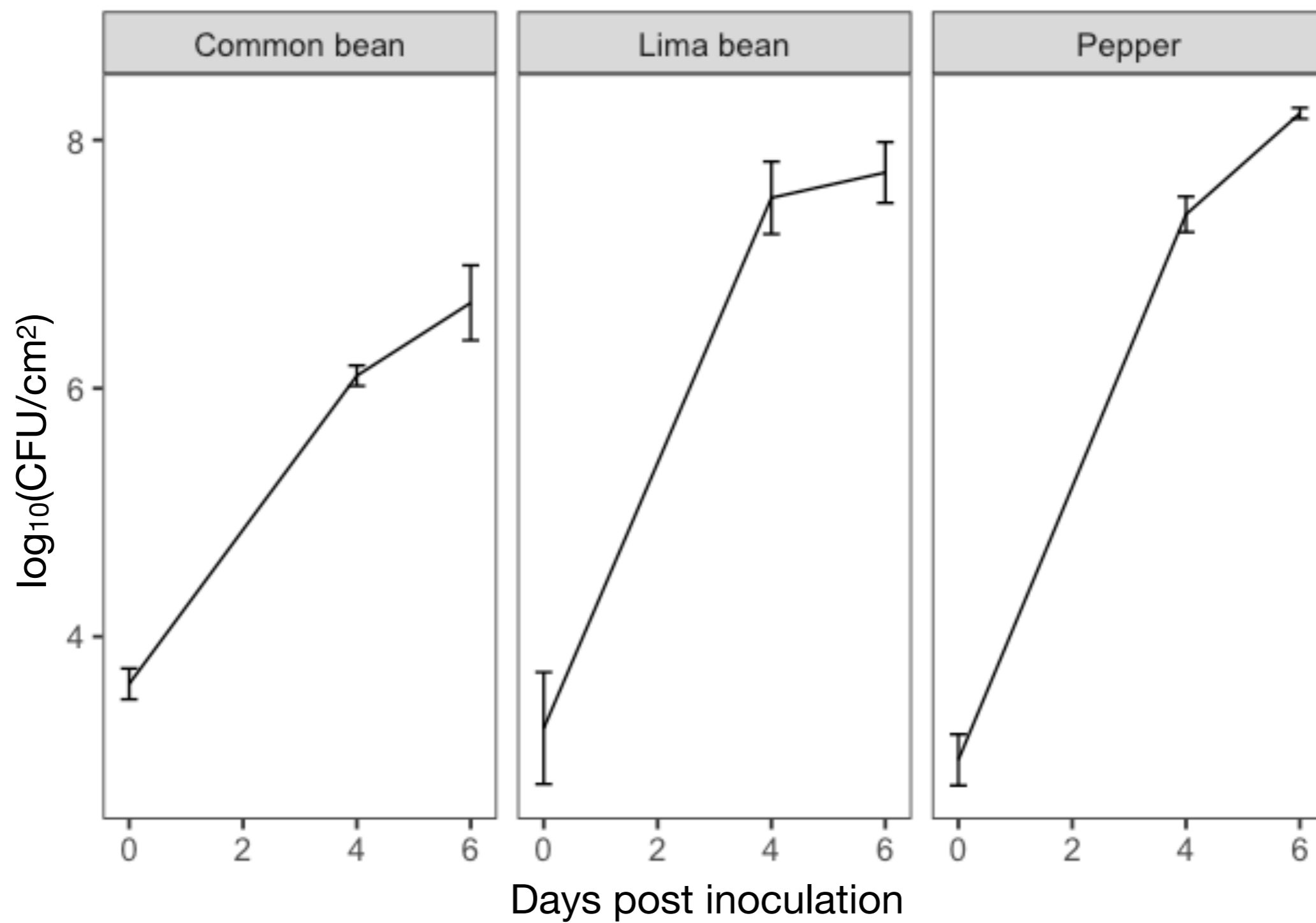

Supplement: S1 Fig — The vertical bars represent the standard deviation of the mean. (PDF) [file pone.0239998.s001.pdf]

S2 Fig

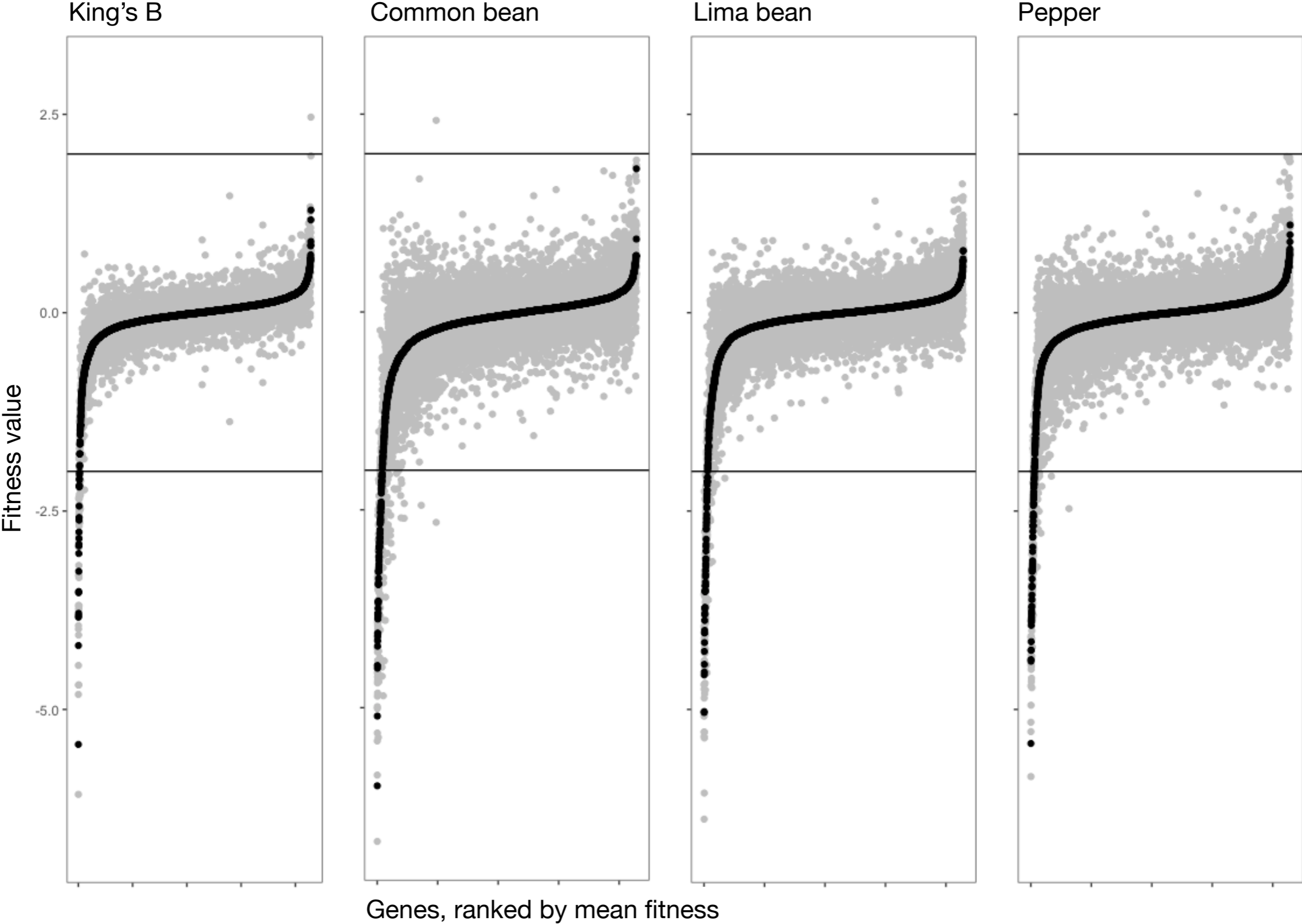

Supplement: S2 Fig — Fitness values for independent replicate experiments are shown in grey, while mean fitness values are shown in black. Gene fitness value is calculated as the log2 of the ratio of the barcode counts following growth in a given setting compared to the barcode counts before inoculation. Black lines indicated at fitness values of -2 and +2 are used to reveal strong phenotypes; for example a value of -2 indicates that mutants are 25% as fit as the typical strain in the mutant library. In each dataset, fitness values < -2 or > +2 are more than three standard deviations from the mean (approximately 0). (PDF) [file pone.0239998.s002.pdf]

### S3 Fig

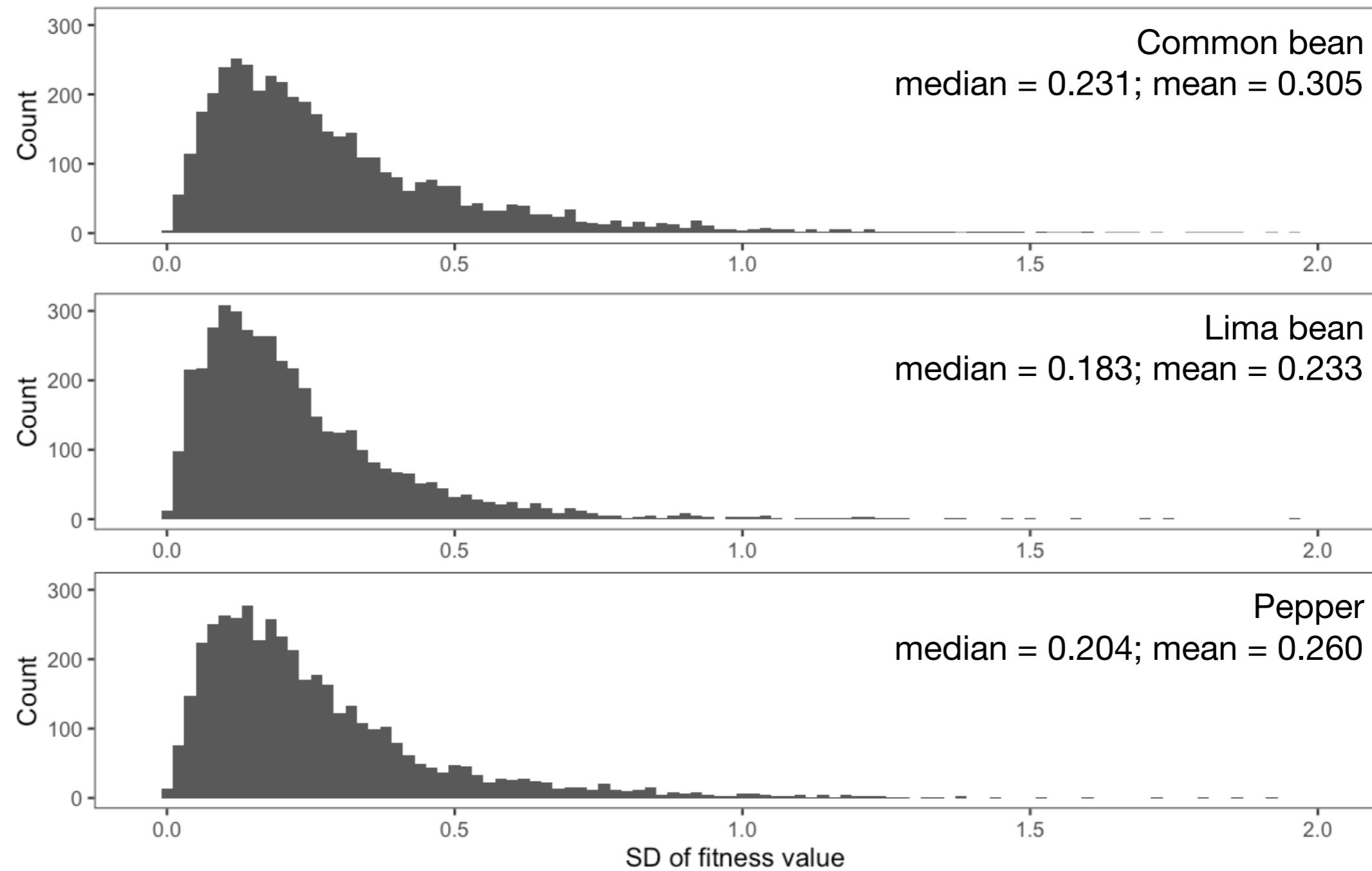

Supplement: S3 Fig — These distributions are similar for the three plant species. (PDF) [file pone.0239998.s003.pdf]

## S4 Fig

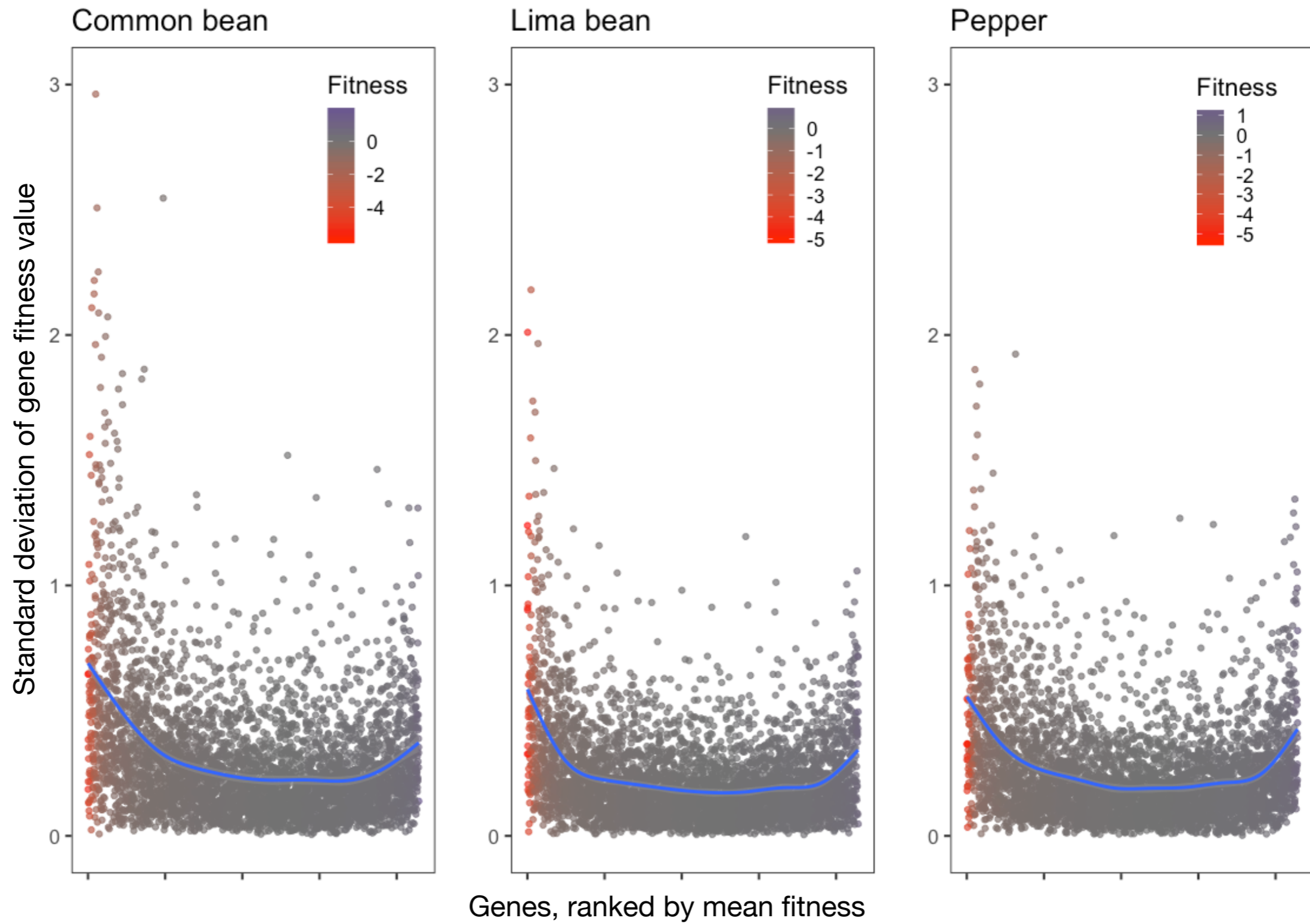

Supplement: S4 Fig — For each host plant, the standard deviations of gene fitness values are ranked by average gene fitness, and are highest on average for genes with very low or high fitness value. A generalized additive model (GAM) was used to fit the regression lines. (PDF) [file pone.0239998.s004.pdf]
