# Supplementary material for: Distinctiveness of genes contributing to growth of Pseudomonas syringae in diverse host plant species
Source: PLoS One. 2020 Sep 28;15(9):e0239998. doi: 10.1371/journal.pone.0239998 (PMC7521676; doi:10.1371/journal.pone.0239998)
Supplement: S1 Table — Values obtained from mid-log phase cultures following library outgrowth and before inoculation (time0) and from leaves pooled after growth in 100 pots of plants of a particular host (sample). Unique barcodes were calculated as the total that mapped to the genome and for which 3 or more reads were obtained in a given experiment. The total number of genome-mapped barcodes in the library was 281,417. Technical (sequencing) replicates are listed separately (“a” and “b”), and share the same time0 reference sample. For an experiment to pass quality control, the median reads per gene in the sample must be ≥ 50 [12]. (DOCX) [file pone.0239998.s005.docx]

**S1 Table.** **Numbers of unique barcodes per sample and median reads per gene.** Values obtained from mid-log phase cultures following library outgrowth and before inoculation (time0) and from leaves pooled after growth in 100 pots of plants of a particular host (sample). Unique barcodes were calculated as the total that mapped to the genome and for which 3 or more reads were obtained in a given experiment. The total number of genome-mapped barcodes in the library was 281,417. Technical (sequencing) replicates are listed separately (“a” and “b”), and share the same time0 reference sample. For an experiment to pass quality control, the median reads per gene in the sample must be ≥ 50 [1].

| Experiment | Unique barcodes at time 0 | Unique barcodes in sample | Recovery (%) | Median reads/gene at time 0 | Median reads/gene in sample |
| --- | --- | --- | --- | --- | --- |
| KB_1 | 187,482 | 192,078 | >100^a^ | 278 | 150 |
| KB_2 | 197,089 | 226,857 | >100^a^ | 378.5 | 251 |
| *P.vulgaris*_1a | 218,149 | 149,311 | 68.4 | 409.5 | 163 |
| *P.vulgaris*_1b | 218,149 | 151,211 | 69.3 | 409.5 | 155 |
| *P.vulgaris*_2 | 214,346 | 156,416 | 73.0 | 390 | 160 |
| *P.vulgaris*_3a | 222,473 | 185,183 | 83.2 | 397 | 203 |
| *P.vulgaris*_3b | 222,473 | 187,289 | 84.2 | 397 | 216 |
| *P.lunatus*_1a | 238,593 | 197,334 | 82.7 | 511.5 | 245 |
| *P.lunatus*_1b | 238,593 | 198,378 | 83.1 | 511.5 | 252 |
| *P.lunatus*_2a | 211,661 | 182,104 | 86.0 | 356 | 175 |
| *P.lunatus*_2b | 211,661 | 181,831 | 85.9 | 356 | 166 |
| *P.lunatus*_3a | 208,924 | 199,475 | 95.5 | 368 | 214 |
| *P.lunatus*_3b | 208,924 | 196,817 | 94.2 | 368 | 203 |
| *C.annuum*_1a | 220,024 | 163,572 | 74.3 | 398 | 198 |
| *C.annuum*_1b | 220,024 | 166,370 | 75.6 | 398 | 206 |
| *C.annuum*_2a | 208,449 | 194,666 | 93.4 | 383.5 | 212 |
| *C.annuum*_2b | 208,449 | 201,173 | 96.5 | 383.5 | 220 |
| *C.annuum*_3a | 220,860 | 198,541 | 89.9 | 406 | 219 |
| *C.annuum*_3b | 220,860 | 204,372 | 92.5 | 406 | 239.5 |

^a^More unique barcodes sequenced at the end of an experiment indicates that additional unique barcodes were present at the threshold abundance in the condition but were below the threshold at the start of the experiment (time0).

1. Wetmore KM, Price MN, Waters RJ, Lamson JS, He J, Hoover CA, et al. Rapid quantification of mutant fitness in diverse bacteria by sequencing randomly bar-coded transposons. MBio. 2015;6: 1–15. doi:10.1128/mBio.00306-15
