# Supplementary material for: Distinctiveness of genes contributing to growth of Pseudomonas syringae in diverse host plant species
Source: PLoS One. 2020 Sep 28;15(9):e0239998. doi: 10.1371/journal.pone.0239998 (PMC7521676; doi:10.1371/journal.pone.0239998)
Supplement: S2 Table — The fitness value cutoff of < -1 is inclusive of the cutoff < -2, although individual genes may appear significant in different categories. These totals are shown as Venn diagrams in Fig 3. (DOCX) [file pone.0239998.s006.docx]

**S2 Table.** **Unique and shared gene loci among the three hosts tested having average fitness values less than -2 or -1.** The fitness value cutoff of < -1 is inclusive of the cutoff < -2, although individual genes may appear significant in different categories. These totals are shown as Venn diagrams in Fig 3.

| **Host[s]** | **Fitness cutoff** | **Total** | **Genes** |
| --- | --- | --- | --- |
| Common bean  Lima bean  Pepper | -2 | 34 | Psyr_0033 Psyr_0034 Psyr_0167 Psyr_0219 Psyr_0473 Psyr_0474 Psyr_0529 Psyr_0531 Psyr_0917 Psyr_0918 Psyr_1212 Psyr_1257 Psyr_1269 Psyr_1350 Psyr_1373 Psyr_1613 Psyr_1663 Psyr_1668 Psyr_1669 Psyr_1748 Psyr_1983 Psyr_1984 Psyr_1985 Psyr_2980 Psyr_3008 Psyr_3958 Psyr_4130 Psyr_4270 Psyr_4369 Psyr_4407 Psyr_4580 Psyr_4581 Psyr_4609 Psyr_4991 |
| Common bean  Lima bean  Pepper | -1 | 90 | Psyr_0014 Psyr_0033 Psyr_0034 Psyr_0167 Psyr_0219 Psyr_0377 Psyr_0378 Psyr_0454 Psyr_0469 Psyr_0473 Psyr_0474 Psyr_0528 Psyr_0529 Psyr_0531 Psyr_0532 Psyr_0557 Psyr_0704 Psyr_0826 Psyr_0827 Psyr_0846 Psyr_0847 Psyr_0848 Psyr_0914 Psyr_0915 Psyr_0917 Psyr_0918 Psyr_0919 Psyr_0920 Psyr_0936 Psyr_0951 Psyr_1056 Psyr_1190 Psyr_1197 Psyr_1198 Psyr_1200 Psyr_1205 Psyr_1206 Psyr_1208 Psyr_1210 Psyr_1211 Psyr_1212 Psyr_1213 Psyr_1216 Psyr_1257 Psyr_1269 Psyr_1350 Psyr_1373 Psyr_1408 Psyr_1410 Psyr_1544 Psyr_1613 Psyr_1663 Psyr_1668 Psyr_1669 Psyr_1747 Psyr_1748 Psyr_1983 Psyr_1984 Psyr_1985 Psyr_2613 Psyr_2980 Psyr_3008 Psyr_3179 Psyr_3636 Psyr_3637 Psyr_3883 Psyr_3958 Psyr_4091 Psyr_4130 Psyr_4132 Psyr_4270 Psyr_4340 Psyr_4341 Psyr_4361 Psyr_4362 Psyr_4369 Psyr_4407 Psyr_4408 Psyr_4566 Psyr_4580 Psyr_4581 Psyr_4609 Psyr_4686 Psyr_4687 Psyr_4852 Psyr_4893 Psyr_4894 Psyr_4896 Psyr_4991 Psyr_5132 |
| Common bean  Lima bean | -2 | 17 | Psyr_0014 Psyr_0377 Psyr_0378 Psyr_0557 Psyr_0704 Psyr_0826 Psyr_0915 Psyr_1056 Psyr_1408 Psyr_1410 Psyr_1614 Psyr_3179 Psyr_3883 Psyr_4408 Psyr_4683 Psyr_4687 Psyr_4852 |
| Common bean  Lima bean | -1 | 25 | Psyr_0025 Psyr_0534 Psyr_0579 Psyr_0916 Psyr_0923 Psyr_1054 Psyr_1055 Psyr_1121 Psyr_1401 Psyr_1614 Psyr_1914 Psyr_2077 Psyr_2461 Psyr_3174 Psyr_3199 Psyr_3287 Psyr_4018 Psyr_4158 Psyr_4194 Psyr_4683 Psyr_4684 Psyr_4843 Psyr_5065 Psyr_5130 Psyr_5133 |
| Common bean  Pepper | -2 | 15 | Psyr_0469 Psyr_0576 Psyr_0846 Psyr_0847 Psyr_0848 Psyr_1217 Psyr_1544 Psyr_2613 Psyr_4132 Psyr_4133 Psyr_4134 Psyr_4893 Psyr_4894 Psyr_4896 Psyr_4897 |
| Common bean  Pepper | -1 | 17 | Psyr_0385 Psyr_0386 Psyr_0576 Psyr_1191 Psyr_1196 Psyr_1215 Psyr_1217 Psyr_1218 Psyr_3193 Psyr_4133 Psyr_4134 Psyr_4144 Psyr_4512 Psyr_4844 Psyr_4897 Psyr_4940 Psyr_5072 |
| Lima bean  Pepper | -2 | 2 | Psyr_1198 Psyr_4362 |
| Lima bean  Pepper | -1 | 2 | Psyr_0555 Psyr_1195 |
| Common bean | -2 | 17 | Psyr_0454 Psyr_0528 Psyr_0914 Psyr_0919 Psyr_0920 Psyr_0936 Psyr_0951 Psyr_1196 Psyr_2612 Psyr_3637 Psyr_4116 Psyr_4144 Psyr_4340 Psyr_4341 Psyr_4684 Psyr_4686 Psyr_4740 |
| Common bean | -1 | 57 | Psyr_0103 Psyr_0201 Psyr_0268 Psyr_0475 Psyr_0533 Psyr_0550 Psyr_0758 Psyr_0831 Psyr_1097 Psyr_1109 Psyr_1247 Psyr_1395 Psyr_1417 Psyr_1419 Psyr_1487 Psyr_1542 Psyr_1588 Psyr_1733 Psyr_1751 Psyr_1907 Psyr_1998 Psyr_2221 Psyr_2264 Psyr_2396 Psyr_2474 Psyr_2501 Psyr_2557 Psyr_2601 Psyr_2612 Psyr_3028 Psyr_3427 Psyr_3552 Psyr_3597 Psyr_3667 Psyr_3675 Psyr_3676 Psyr_3678 Psyr_3690 Psyr_3698 Psyr_3791 Psyr_3889 Psyr_4015 Psyr_4044 Psyr_4069 Psyr_4116 Psyr_4125 Psyr_4136 Psyr_4143 Psyr_4224 Psyr_4740 Psyr_4754 Psyr_4774 Psyr_4882 Psyr_4895 Psyr_5053 Psyr_5067 Psyr_5129 |
| Lima bean | -2 | 5 | Psyr_0025 Psyr_0532 Psyr_0923 Psyr_1914 Psyr_4018 |
| Lima bean | -1 | 28 | Psyr_0202 Psyr_0259 Psyr_0435 Psyr_0478 Psyr_0524 Psyr_0796 Psyr_0822 Psyr_1053 Psyr_1057 Psyr_1058 Psyr_1059 Psyr_1060 Psyr_1061 Psyr_1062 Psyr_1063 Psyr_1140 Psyr_1667 Psyr_1749 Psyr_2462 Psyr_3146 Psyr_3684 Psyr_3691 Psyr_4008 Psyr_4019 Psyr_4100 Psyr_4627 Psyr_4842 Psyr_4898 |
| Pepper | -2 | 7 | Psyr_0385 Psyr_0386 Psyr_1190 Psyr_1191 Psyr_1208 Psyr_1210 Psyr_4566 |
| Pepper | -1 | 8 | Psyr_1111 Psyr_2080 Psyr_2245 Psyr_2543 Psyr_2617 Psyr_3459 Psyr_3835 Psyr_5135 |
