# Supplementary material for: Distinctiveness of genes contributing to growth of Pseudomonas syringae in diverse host plant species
Source: PLoS One. 2020 Sep 28;15(9):e0239998. doi: 10.1371/journal.pone.0239998 (PMC7521676; doi:10.1371/journal.pone.0239998)
Supplement: S3 Table — Fitness values in KB are included for comparison. This table does not include 37 genes whose average fitness values differed between plant species but whose effect was small, having fitness values greater than -0.5 and less than +0.5 in all host plant species. (DOCX) [file pone.0239998.s007.docx]

**S3 Table.** **Genes having a differential fitness contribution to growth in three plant species by a Kruskal-Wallis rank sum test (p < 0.05).** Fitness values in KB are included for comparison. This table does not include 37 genes whose average fitness values differed between plant species but whose effect was small, having fitness values greater than -0.5 and less than +0.5 in all host plant species.

| Locus | Name | Description | Classification | Average fitness | | | |
| --- | --- | --- | --- | --- | --- | --- | --- |
|  |  |  |  | KB | Common bean | Lima bean | Pepper |
| Psyr_0025 | aroE | shikimate dehydrogenase | Amino acid metabolism and transport | -0.25 | -1.62 | -2.95 | -0.31 |
| Psyr_4133 | hisD | histidinol dehydrogenase | Amino acid metabolism and transport | 0.12 | -2.99 | -0.83 | -3.80 |
| Psyr_4896 | hisH | imidazole glycerol phosphate synthase subunit hisH | Amino acid metabolism and transport | -0.10 | -2.20 | -1.06 | -3.23 |
| Psyr_4852 |  | D-3-phosphoglycerate dehydrogenase | Amino acid metabolism and transport | -0.13 | -2.94 | -3.40 | -1.95 |
| Psyr_0758 | scrB | beta-fructofuranosidase | Carbohydrate metabolism and transport | 0.05 | -1.66 | -0.94 | -0.52 |
| Psyr_2188 |  | Histidine kinase, HAMP region:Bacterial chemotaxis sensory transducer:CHASE3 | Chemosensing & chemotaxis | -0.23 | -0.88 | 0.18 | -0.15 |
| Psyr_4687 | bioB | biotin synthase | Cofactor metabolism | -2.94 | -2.86 | -2.24 | -1.63 |
| Psyr_4684 | bioC | biotin synthesis protein BioC | Cofactor metabolism | -2.76 | -2.85 | -1.79 | -0.87 |
| Psyr_4686 | bioF | 8-amino-7-oxononanoate synthase | Cofactor metabolism | -3.03 | -2.62 | -1.99 | -1.30 |
| Psyr_2098 | cobA | uroporphyrinogen-III C-methyltransferase | Cofactor metabolism | 0.27 | -0.56 | 0.08 | -0.23 |
| Psyr_0847 | ilvH | acetolactate synthase, small subunit | Cofactor metabolism | 0.08 | -2.45 | -1.44 | -3.89 |
| Psyr_0846 | ilvI | acetolactate synthase, large subunit | Cofactor metabolism | -0.18 | -3.10 | -1.92 | -3.70 |
| Psyr_3487 | flgA | flagellar protein FlgA | Flagellar synthesis and motility | 0.14 | 0.27 | 0.02 | 0.78 |
| Psyr_0487 | gshB | glutathione synthase | Glutathione metabolism | -1.54 | 1.81 | 0.78 | -0.34 |
| Psyr_1716 |  | conserved hypothetical protein | Hypothetical | 0.26 | -0.87 | -0.40 | 0.00 |
| Psyr_2543 |  | conserved hypothetical protein | Hypothetical | 0.57 | -0.07 | 0.34 | -1.05 |
| Psyr_3107 |  | conserved hypothetical protein | Hypothetical | -0.18 | -0.54 | 0.20 | 0.44 |
| Psyr_3172 |  | Glycosyl transferase, family 3 | Hypothetical | -0.04 | 0.52 | -0.30 | 0.11 |
| Psyr_3611 |  | Protein of unknown function DUF815 | Hypothetical | 0.22 | 0.68 | -0.56 | 0.10 |
| Psyr_3889 |  | conserved hypothetical protein | Hypothetical | -0.01 | -1.39 | -0.06 | 0.52 |
| Psyr_0014 |  | lipid A biosynthesis acyltransferase | LPS synthesis and transport | -0.53 | -2.66 | -2.08 | -1.44 |
| Psyr_3369 |  | Twin-arginine translocation pathway signal:Tat-translocated enzyme:Dyp-type peroxidase | Oxidative stress tolerance (Antioxidant enzyme) | -0.10 | -0.55 | 0.22 | -0.25 |
| Psyr_2621 | pseB | Secretion protein HlyD | Phytotoxin synthesis and transport | -0.06 | 0.25 | -0.03 | -0.53 |
| Psyr_2601 | salA | regulatory protein, LuxR | Phytotoxin synthesis and transport | -0.37 | -1.21 | -0.23 | -0.68 |
| Psyr_1702 | sylA | regulatory protein, LuxR | Phytotoxin synthesis and transport | -0.26 | -0.54 | -0.10 | 0.18 |
| Psyr_2614 | sypA | Amino acid adenylation | Phytotoxin synthesis and transport | 0.03 | -0.85 | -0.15 | -0.69 |
| Psyr_2612 | syrP | syrP protein, putative | Phytotoxin synthesis and transport | 0.04 | -2.07 | -0.02 | -0.70 |
| Psyr_4158 | eftA | conserved hypothetical protein | Plant-associated proteins | -0.39 | -1.42 | -1.01 | -0.09 |
| Psyr_1061 | alg44 | alginate biosynthesis protein Alg44 | Polysaccharide synthesis and regulation | 0.02 | -0.72 | -1.28 | -0.34 |
| Psyr_1062 | alg8 | alginate biosynthesis protein Alg8 | Polysaccharide synthesis and regulation | -0.04 | -0.70 | -1.22 | -0.33 |
| Psyr_0937 | algA-1 | mannose-6-phosphate isomerase, type 2 / mannose-1-phosphate guanylyltransferase (GDP) | Polysaccharide synthesis and regulation | -0.30 | -0.70 | -0.47 | -0.16 |
| Psyr_1053 | algF | alginate biosynthesis protein AlgF | Polysaccharide synthesis and regulation | -0.04 | -0.81 | -1.48 | -0.43 |
| Psyr_1055 | algI | Membrane bound O-acyl transferase, MBOAT | Polysaccharide synthesis and regulation | 0.00 | -1.03 | -1.57 | -0.36 |
| Psyr_1060 | algK | Sel1-like repeat protein | Polysaccharide synthesis and regulation | 0.17 | -0.51 | -1.21 | -0.28 |
| Psyr_0378 | mdoH | Glycosyl transferase, family 2 | Polysaccharide synthesis and regulation | -1.04 | -3.80 | -4.04 | -1.08 |
| Psyr_3636 | wbpM | Polysaccharide biosynthesis protein CapD | Polysaccharide synthesis and regulation | -0.45 | -1.76 | -1.48 | -1.10 |
| Psyr_3161 | aprD | Type I secretion system ATPase, PrtD | Secretion/Efflux/Export | 0.39 | 0.11 | 0.38 | -0.61 |
| Psyr_4009 | oprM | RND efflux system, outer membrane lipoprotein, NodT | Secretion/Efflux/Export | 0.02 | -0.52 | -0.95 | 0.20 |
| Psyr_0831 | cbrB-1 | Two-component response regulator CbrB | Signal transduction mechanisms | -1.45 | -1.71 | -0.98 | 0.17 |
| Psyr_4069 | colS | ATP-binding region, ATPase-like:Histidine kinase, HAMP region:Histidine kinase A, N-terminal | Signal transduction mechanisms | -0.08 | -1.21 | -0.57 | -0.38 |
| Psyr_4138 |  | Toluene tolerance | Stress resistance | -0.22 | -0.20 | -0.59 | 0.28 |
| Psyr_3698 | gacS | Response regulator receiver:ATP-binding region, ATPase-like:Histidine kinase, HAMP region:Histidine kinase A, N-terminal:Hpt | Transcriptional regulation | 0.07 | -1.47 | -0.44 | -0.85 |
| Psyr_4239 | dppB | Binding-protein-dependent transport systems inner membrane component | Transport (peptides) | -0.09 | -0.58 | -0.33 | -0.07 |
| Psyr_4240 | dppC | Binding-protein-dependent transport systems inner membrane component | Transport (peptides) | 0.14 | -0.57 | -0.34 | 0.01 |
| Psyr_1218 | hrpK1 | type III helper protein HrpK1 | Type III secretion system | 0.08 | -1.28 | -0.75 | -1.67 |
| Psyr_0914 | wpbZ | Glycosyl transferase, group 1 |  | -0.19 | -2.08 | -1.83 | -1.32 |
| Psyr_0915 |  | NAD-dependent epimerase/dehydratase |  | -0.21 | -3.19 | -3.51 | -1.36 |
| Psyr_1419 |  | preQ(0) biosynthesis protein QueC |  | -0.65 | -1.85 | -0.87 | -0.10 |
| Psyr_4844 |  | HAD-superfamily hydrolase, subfamily IB (PSPase-like):HAD-superfamily subfamily IB hydrolase, hypothetical 2 |  | -0.10 | -1.00 | -0.30 | -1.69 |
| Psyr_4886 |  | Peptidase M23B |  | -0.05 | -0.53 | -0.30 | -0.07 |
