# Supplementary material for: Distinctiveness of genes contributing to growth of Pseudomonas syringae in diverse host plant species
Source: PLoS One. 2020 Sep 28;15(9):e0239998. doi: 10.1371/journal.pone.0239998 (PMC7521676; doi:10.1371/journal.pone.0239998)
Supplement: S4 Table — Fitness values in KB are included for comparison. (DOCX) [file pone.0239998.s008.docx]

**S4 Table.** Genes having a differential fitness contribution to growth in three plant species by a Kruskal-Wallis rank sum test (p < 0.05), but whose effect was small, having fitness values greater than -0.5 and less than +0.5 in all host plant species. Fitness values in KB are included for comparison.

| Locus | Name | Description | Classification | Average fitness | | | |
| --- | --- | --- | --- | --- | --- | --- | --- |
|  |  |  |  | KB | Common bean | Lima bean | Pepper |
| Psyr_1074 | aapM | amino acid ABC transporter membrane protein 2, PAAT family | Amino acid metabolism and transport | 0.17 | 0.10 | 0.28 | -0.06 |
| Psyr_3876 | hisM | amino acid ABC transporter membrane protein 2, PAAT family | Amino acid metabolism and transport | -0.04 | 0.23 | 0.08 | -0.09 |
| Psyr_2715 |  | Major facilitator superfamily | Carbohydrate metabolism and transport | 0.02 | 0.30 | -0.16 | 0.12 |
| Psyr_3406 | aer-2 | PAS | Chemosensing & chemotaxis | -0.06 | -0.29 | 0.05 | -0.13 |
| Psyr_2995 | treY | maltooligosyl trehalose synthase | Compatible solute synthesis | 0.02 | -0.45 | -0.18 | 0.01 |
| Psyr_1481 | ppa-2 | Inorganic diphosphatase | Energy generation | -0.09 | -0.06 | 0.03 | -0.20 |
| Psyr_1770 |  | Enoyl-CoA hydratase/isomerase | Fatty acid metabolism | -0.07 | -0.06 | -0.27 | 0.30 |
| Psyr_3456 | fliG | Flagellar motor switch protein FliG | Flagellar synthesis and motility | 0.09 | -0.24 | 0.04 | 0.38 |
| Psyr_3613 |  | Glutathione peroxidase | Glutathione metabolism | 0.12 | 0.39 | -0.19 | 0.08 |
| Psyr_0332 |  | hypothetical protein | Hypothetical | -0.06 | 0.21 | -0.10 | 0.05 |
| Psyr_1137 |  | Protein of unknown function UPF0153 | Hypothetical | 0.00 | -0.12 | 0.26 | 0.12 |
| Psyr_1407 |  | Protein of unknown function DUF28 | Hypothetical | -0.07 | 0.41 | 0.07 | -0.31 |
| Psyr_1533 |  | hypothetical protein | Hypothetical | 0.16 | -0.39 | -0.11 | 0.15 |
| Psyr_2339 |  | hypothetical protein | Hypothetical | 0.09 | 0.46 | -0.37 | 0.18 |
| Psyr_2947 |  | hypothetical protein | Hypothetical | -0.22 | 0.47 | -0.01 | -0.39 |
| Psyr_3006 |  | Protein of unknown function DUF419 | Hypothetical | -0.10 | 0.40 | -0.10 | 0.06 |
| Psyr_3066 |  | conserved hypothetical protein | Hypothetical | -0.22 | -0.10 | -0.39 | 0.27 |
| Psyr_3740 |  | Protein of unknown function DUF454 | Hypothetical | 0.03 | -0.33 | 0.01 | 0.13 |
| Psyr_3798 |  | conserved domain protein | Hypothetical | 0.01 | 0.16 | 0.01 | -0.09 |
| Psyr_4248 |  | hypothetical protein | Hypothetical | 0.01 | 0.08 | 0.14 | -0.01 |
| Psyr_5112 |  | conserved hypothetical protein | Hypothetical | -0.13 | 0.03 | -0.06 | 0.16 |
| Psyr_1318 | ppc | Phosphoenolpyruvate carboxylase | Organic acid metabolism and transport | 0.15 | -0.18 | 0.22 | 0.09 |
| Psyr_1705 | sylD | Amino acid adenylation | Phytotoxin synthesis and transport | 0.03 | -0.32 | -0.02 | -0.06 |
| Psyr_4615 |  | Spermidine/putrescine ABC transporter ATP-binding subunit | Polyamine metabolism and transport | -0.09 | -0.15 | 0.10 | 0.20 |
| Psyr_3956 | mucB | sigma E regulatory protein, MucB/RseB | Polysaccharide synthesis and regulation | -0.04 | 0.06 | 0.01 | 0.33 |
| Psyr_3236 | dhcR | transcriptional regulator, LysR family | QAC metabolism and transport | 0.32 | -0.12 | 0.06 | 0.29 |
| Psyr_2576 | syfA | Amino acid adenylation | Secondary metabolism | 0.10 | -0.03 | 0.10 | 0.31 |
| Psyr_2864 |  | RND efflux system, outer membrane lipoprotein, NodT | Secretion/Efflux/Export | 0.14 | -0.11 | -0.01 | -0.28 |
| Psyr_3989 | xaxA | hypothetical protein | Special | -0.02 | 0.15 | 0.08 | -0.02 |
| Psyr_4273 | cstA | Carbon starvation protein CstA | Stress resistance | 0.05 | -0.05 | 0.04 | 0.15 |
| Psyr_0986 | rsmC | 16S rRNA m(2)G 1207 methyltransferase |  | -0.19 | -0.37 | 0.00 | 0.37 |
| Psyr_0929 |  | Glycosyl transferase, family 2 |  | 0.11 | -0.20 | -0.07 | -0.02 |
| Psyr_0989 |  | Lysine exporter protein (LYSE/YGGA) |  | 0.06 | -0.25 | -0.03 | 0.32 |
| Psyr_3859 |  | Purine nucleoside permease |  | -0.01 | -0.23 | -0.08 | 0.04 |
| Psyr_4631 |  | PrkA serine kinase |  | 0.07 | -0.09 | -0.03 | 0.07 |
| Psyr_5082 |  | Band 7 protein |  | -0.02 | -0.01 | -0.24 | 0.11 |
| Psyr_5111 |  | dTDP-glucose 4,6-dehydratase |  | -0.06 | 0.08 | -0.03 | -0.13 |
