# Supplementary material for: Distinctiveness of genes contributing to growth of Pseudomonas syringae in diverse host plant species
Source: PLoS One. 2020 Sep 28;15(9):e0239998. doi: 10.1371/journal.pone.0239998 (PMC7521676; doi:10.1371/journal.pone.0239998)
Supplement: S5 Table — (DOCX) [file pone.0239998.s009.docx]

**S5 Table.** **Strains used in this study.**

| Strains | Genotype | Reference |
| --- | --- | --- |
| *P. syringae* B728a | Wild type strain (Rif^R^) | [1] |
| *P. syringae* B728a | Whole genome barcoded *mariner* transposon library (Rif^R^ Kan^R^) | [2] |

1. Loper JE, Lindow SE. Lack of evidence for *in situ* fluorescent pigment production by *Pseudomonas syringae* pv. *syringae* on bean leaf surfaces. Phytopathology. 1987;77: 1449–1454. doi:10.1094/Phyto-77-1449

2. Helmann TC, Deutschbauer AM, Lindow SE. Genome-wide identification of *Pseudomonas syringae* genes required for fitness during colonization of the leaf surface and apoplast. Proc Natl Acad Sci. 2019;116: 18900–18910. doi:10.1073/pnas.1908858116
